# Recent advances in pharmacotherapy of glaucoma

**DOI:** 10.4103/0253-7613.44151

**Published:** 2008-10

**Authors:** S. K. Gupta, Galpalli Niranjan D., S. S. Agrawal, Sushma Srivastava, Rohit Saxena

**Affiliations:** Delhi Institute of Pharmaceutical Sciences and Research, New Delhi, India; 1 Dr. Rajendra Prasad Center for Ophthalmic Sciences, AIIMS, New Delhi, India

**Keywords:** Adrenergic blockers, carbonic anhydrase inhibitors, cholinergic agonists, intraocular pressure, prostaglandin analogs

## Abstract

Glaucoma is a slow progressive degeneration of the retinal ganglion cells (RGCs) and the optic nerve axons, leading to irreversible blindness if left undiagnosed and untreated. Although increased intraocular pressure is a major risk factor of glaucoma, other factors include increased glutamate levels, alterations in nitric oxide (NO) metabolism, vascular alterations and oxidative damage caused by reactive oxygen species. Glaucoma is the second leading cause of blindness globally, accounting for 12.3% of the total blindness. Glaucoma has been broadly classified as primary or secondary open-angle or angle-closure glaucoma. The primary goal in management of glaucoma is to prevent the risk factor, especially elevated intraocular pressure (IOP), using medications, laser therapy or conventional surgery. The first-line treatment of glaucoma usually begins with the use of a topical selective or nonselective blocker or a prostaglandin analog. Second-line drugs of choice include alpha-agonists and topical carbonic anhydrase inhibitors. Cholinergic agonists are considered third-line treatment options. When a single therapy is not sufficient to lower the IOP, a combination therapy is indicated. To enhance the patient compliance, drug delivery systems like electronic devices, ocular inserts, tansdermal and mechanical drug delivery systems have been developed. Use of viscoelastic agents in ophthalmic formulations, emulsions and soluble ophthalmic drug inserts (SODI) enhance patience compliance and ocular drug delivery in patients in long-term glaucoma therapy. For patients who do not respond to antiglaucoma medications, laser trabeculoplasty and incisional surgery are recommended. Several nutrients and botanicals hold promise for the treatment of glaucoma, but most studies are preliminary, and larger, controlled studies are required. Future directions for the development of a novel therapy glaucoma may target glutamate inhibition, NMDA receptor blockade, exogenously applied neurotrophins, open channel blockers, antioxidants, protease inhibitors and gene therapy.

## Introduction

Glaucoma is characterized by slow progressive degeneration of the retinal ganglion cells (RGCs) and the optic nerve axons, leading to increasing deterioration of the visual field. If untreated, the condition can lead to irreversible blindness.[1]

Glaucoma represents a common pathway for different eye conditions, many of which are associated with elevated intraocular pressure (IOP). The involvement of excitatory and inhibitory nerve transmitters like glutamates, gamma-amino butyric acid (GABA), glycine and apoptosis can be implicated as a mechanism of progression of glaucoma. Early detection and treatment can slow, or even halt the progression of the disease. However, glaucoma often progresses despite lowering of the IOP to acceptable or normal levels.[2]

### Magnitude

Glaucoma is the second leading cause of blindness. Worldwide, it is estimated that about 66.8 million people have visual impairment from glaucoma, with 6.7 million suffering from blindness. The prevalence of glaucoma increases with age. Two percent of the population older than 40 years of age and five to nine percent of those older than 65 years have glaucoma.[3,4] It is estimated that there will be 60.5 million people with OAG (open angle glaucoma) and ACG (angle closure glaucoma) in 2010, which will increase to 79.6 million by 2020. Of these, 74% will have OAG. From 2010 to 2020, the most detectable change in glaucoma worldwide will be an increase of the incidence of glaucoma in India. As the proportion of those over age 40 increases, the proportional increase in glaucoma will challenge our resources and ingenuity.[5]

### Risk factors for the development of glaucoma

Although increased intraocular pressure is the major risk factor for primary open angle glaucoma (POAG), other factors such as increased glutamate levels, alterations in nitric oxide (NO) metabolism, vascular alterations and oxidative damage caused by reactive oxygen species[6] are also involved [Table 1].

**Table 1:** Risk factors for glaucoma

*Factors with strong association with glaucoma*	
Elevated IOP	High IOP is the most important factor for the development of glaucoma.
Family history of glaucoma	The mode of inheritance for POAG is polygenic.
Race	The risk of developing glaucoma is 4.3 times higher in Afro-Caribbean's than in white Americans.
Advanced age	The incidence of POAG is higher in the elderly than in the younger patients.
Corneal thickness	Patients with corneal thickness greater than 588 mm are less likely to progress to POAG.
Factors with moderate association with glaucoma Sex	Females are at greater risk of Normal Tension Glaucoma (2 : 1) and chronic angle closure glaucoma (4 : 1)
Myopia	Increased association glaucoma in myopic patients
Factors with weak association with glaucoma	
Diabetes	Micro-angiopathy may be involved in pathogenesis glaucoma.
Migraine	May be at higher risk for the development of NTG.
Systemic hypertension	Some studies have shown association of raised IOP with increase in blood pressure.

IOP = Intraocular pressure, POAG = Primary open angle glaucoma and NTG = Normal tension glaucoma

## Types of Glaucoma

There are various types of glaucoma. These are:

### Primary open angle glaucoma

It is the most common form of glaucoma throughout world, accounting for about two-thirds of cases.[7] The anterior chamber is deep and there is reduced aqueous outflow through the trabecular meshwork, which leads to a rise in IOP.

### Normal tension glaucoma

It is believed to account for 30% of the glaucoma cases in Western countries and over two-thirds of the cases in Japan. Its incidence in the Indian population is generally considered low. Here, the IOP is within the normal range; however, there may be poor blood flow to the optical disc or increased susceptibility to disc damage at lower IOP.

### Primary angle closure glaucoma

It accounts for nearly 50% of the cases in India. The rise in IOP is caused by closure of the anterior chamber angle. This may be either acute or chronic.

### Secondary open angle or angle closure glaucoma

This is caused by substances mechanically blocking the anterior chamber angle (pigmentary or pseudoexfoliation) or due to an alteration in the structure and function of the trabecular meshwork, owing to trauma, inflammation or ischaemia.[8–10]

### Pharmacotherapy of glaucoma

**Figure 1:**
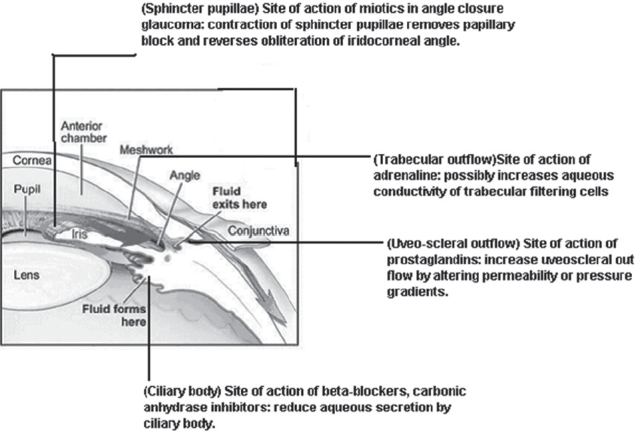
Sites/mechanism of action of different classes of antiglaucoma agents (figure adopted from www.progressiveoptometry.com/_Media/glaucbefo)

Prevention/control of raised intraocular pressure is the primary goal in the management of glaucoma. Modern medicine focuses on three separate targets: IOP, outflow facility, and the retinal ganglion cell, to help us achieve the ultimate goal of therapy and to preserve the visual function in these patients [Table 2, Figure 1]. Many glaucoma medications either reduce or control IOP. But the progression of glaucomatous optic neuropathy can occur even when the IOP is within normal or low range.

**Table 2:** Pharmacotherapy of gluacoma

*Medication*	*Dosage regimen*	*Mechanism of action/Effect on outflow facility β-blockers*	*Side effects*
Betaxolol	0.25 and 0.5% eye drops twice daily	↓ Aqueous production	Stinging upon instillation, reduced side effects compared to timolol
Timolol	0.25 and 0.5% eye drops twice daily	↓ Aqueous production	Systemic: Brochospasm, headache, dizziness, bradycardia, hypotension
			Ocular: Superficial punctate keratitis, ocular pain, corneal anesthesia, diplopia, ptosis
Carteolol	1% eye drops twice daily	↓ Aqueous production	Same as timolol
Levobunalol	0.5% to 1% twice or once a day	↓ Aqueous production	Stinging, bradycardia, hypotension
Cholinergic agonists			
Pilocarpine	0.5 to 8% eye drops 2-4 times daily	↑ Aqueous outflow	Systemic: Salivation, urination
			Ocular: Miosis, follicular conjuctivitis, induced accommodation, retinal detachment, iritis
Adrenergics agonists			
Epinephrine	0.25-2% eye drops twice daily	↑ Aqueous outflow &	Ocular: Blurred vision, conjunctival hyperemia
		↑ Uveoscleral outflow	Systemic: Headache, palpitations, high blood pressure, anxiety
Dipivefrin	0.1% eye drops 2-3 times daily	↑ Aqueous outflow &	Burning, stinging, follicular conjunctivitis, blurry vision, headache
		↑ Uveoscleral outflow	
α-2-Agonists			
Apraclonidine	0.5% and 1% twice daily	↓ Aqueous production &	Tachyphylaxis, allergic blepharoconjuctivitis
		↑Minor increase in aqueous outflow	
Brimonidine	0.2 and 0.5% applied twice daily	↓ Aqueous production &	Irritation, dry mouth, drowsiness
		↑ Minor increase in aqueous outflow	
Systemic carbonic anhydrase inhibitors			
Acetazolamide	Acetazolamide tablets (125 mg and 250 mg four times daily.	↓ Aqueous production	Paresthesia of fingertips and toes, fatigue, depression, kidney stones, thrombocytopenia, agranulocystosis, aplastic anemia
Methazolamide	Methazolamide tablets (25 and 50 mg) 2-3 times daily	↓ Aqueous production	Paresthesia of fingertips and toes, fatigue, depression, kidney stones, thrombocytopenia, agranulocystosis, aplastic anemia
Topical carbonic anhydrase inhibitors			
Dorzolamide	2% ophthalmic solution applied three times daily	↓ Aqueous production	Corneal edema, stinging, burning and itching
Brinzolamide	1% ophthalmic suspension applied three times daily	↓ Aqueous production	Blurred vision, tearing; bitter, dry eyes; headache
Prostaglandin analogs			
Latanoprost	0.005% once daily once daily in the evening	↑ Uveoscleral outflow	Iris pigmentation, mild conjuctival hyperemia, local irritation, cystoid macular edema, increase growth eyelashes
Bimatoprost	0.03% ophthalmic solution once daily in the evening	↑ Uveoscleral outflow	Mild conjunctival hyperemia, Iris pigmentation
Travaprost	0.004% ophthalmic solution once daily in the evening	↑ Uveoscleral outflow	Macular oedema, cystoid macular oedema

The antiglaucoma agents act on the aqueous humor dynamics to reduce the intraocular pressure by different mechanisms.

1. Miotics in angle closure glaucoma act by contraction of sphincter pupillae, which removes papillary block and reverses obliteration of the iridocorneal angle.
2. Miotics in open closure glaucoma act by contraction of the ciliary muscle pulls on the scleral spur and improves trabecular patency.
3. β-blockers and carbonic anhydrase inhibitors reduce aqueous humor secretion by the ciliary body.
4. Prostaglandins increase uveoscleral out flow by altering permeability or pressure gradients.

### Cholinergic agonists

Cholinergics were introduced over 100 years ago and they were the first class of agents used for the treatment of glaucoma.

Direct acting agents work directly on the parasympathetic receptors in the eye, whereas indirect-acting agents inhibit acetylcholinesterase enzyme.

### Pilocarpine

**Figure 2:**
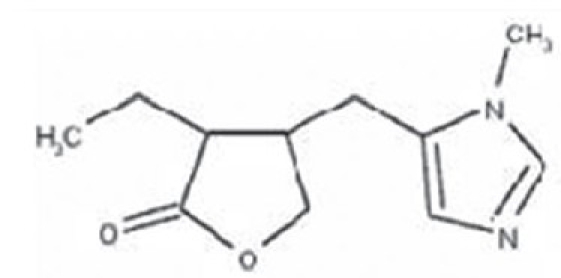
structure of pilocarpine

Pilocarpine [Figure 2] is a muscarinic alkaloid obtained from the leaves of tropical American shrubs, from the genus Pilocarpus. It is the most widely used cholinergic drug for the treatment of glaucoma. It acts by stimulating the muscarinic receptors of the ciliary muscle, which widens the anterior chamber angle, resulting in an increased outflow of aqueous humor through the trabecular meshwork. It is available in concentrations ranging from 0.5 to 10% eye drops. Although it has been available for decades for the treatment of glaucoma, its usage has declined, since more drug options with fewer side effects have become available.

A long term randomized study with combination of pilocarpine 1% and clonidine 0.125% showed an IOP reduction comparable to that achieved with timolol 0.25% twice daily. This combination could be used as a first line therapy in patients wherein β-blockers are contraindicated.[11] The side effects of this drug include miosis, induced accommodation, brow ache, myopic shift, increase risk of retinal detachment and iritis. Pilocarpine produces a reduction in IOP, similar to that of beta-blocking agents, but it is no longer considered a first-line agent for glaucoma treatment, owing to its short duration of action (four to six hours) and side effects.

### Delivery systems for pilocarpine

Pilocarpine gel: Pilocarpine hydrochloride 4% in a highly viscous acrylic vehicle, when applied once daily at bedtime, has been reported to produce significant reduction in IOP for 24 hours.

Membrane-controlled delivery system: This is an insert placed in the cul-de-sac, where it gradually releases pilocarpine at the rate of 20mg/hour, which is roughly equivalent to 2% eye drops. This is effective for seven days and follows zero order kinetics.

Electronic medication alarm device: This device enhances the compliance in glaucoma patients taking pilocarpine.

### Adrenergics

Adrenergic drugs lower intraocular pressure by decreasing blood flow in the ciliary body and through direct receptor-related reduction of aqueous humor production.[12]

### Epinephrine

Epinephrine is a direct acting sympathomimetic amine. Epinephrine stimulates both a and β-adrenoreceptors within the eye. It reduces IOP by increasing the aqueous humor outflow through the trabecular meshwork and uveoscleral pathway. However, recent studies have shown that topical epinephrine did not significantly affect the uveoscleral outflow or the episcleral venous pressure.[13–15] Reduction in IOP ranges from 15-25% for epinephrine, but is accompanied by adverse effects including blurred vision, headache, palpitations, high blood pressure, and anxiety.

### Dipivefrin

Dipivefrin is a prodrug of epinephrine, formed by the diesterification of epinephrine and pivalic acid. The addition of two ester groups (pivaloyl) to the epinephrine molecule enhances its lipophilic character, which helps easy penetration into the anterior chamber. Since it penetrates more easily across the cornea, lesser doses are required. The IOP reduction with dipivefrin is comparable with that of epinephrine. The liberated epinephrine exerts its action by decreasing aqueous production and by enhancing outflow facility. The onset of action with dipivefrin occurs about 30 minutes after treatment, with maximum effect seen at about one hour.

Dipivefrin is more effective than epinephrine; it penetrates the cornea approximately 17 times more than epinephrine.[16] It is indicated for initial therapy or as an adjunct with other ocular hypotensive agents. It produces 20-24% reduction in IOP. Topical dipivefrin 0.1% is useful for lowering IOP in patients intolerant to epinephrine.[17] The most frequent side effects reported with dipivefrin include burning, stinging, follicular conjunctivitis, blurry vision, headache, and allergic reaction.

## Alpha-2-AGONISTS

### Clonidine

Clonidine is the first available a-2 agonist, for the treatment of glaucoma. Clonidine is a lipophilic molecule, which is a relatively selective a-2 adrenoceptor agonist with some a-1 adrenoceptor agonistic activity. It crosses the blood brain barrier and causes systemic hypotension.

### Apraclonidine

Apraclonidine or para aminoclonidine is a derivative a-2 adrenergic agonist. It decrease the aqueous humor secretion and the episcleral venous pressure.[18] It is not recommended as a long-term therapy because of the high incidence of local adverse reactions and tachyphylaxis. It is indicated for short-term use for the prevention or control of post-surgical increases in IOP, and indicated as an adjunctive agent for POAG. Long-term therapy with apraclonidine causes allergic blepharoconjuctivitis.[19] The mean reductions in IOP range from 20% to 27%.[20] Apraclonidine is available as 0.5 and 1% concentrations, to be applied twice daily.

### Brimonidine

Brimonidine is the a-2 agonist of choice in glaucoma treatment, which acts by decreasing the aqueous humor secretion and increasing the uveoscleral outflow. It does not cross the blood-brain barrier and is 30 times more selective for the a-2-adrenergic receptor than apraclonidine.[21] Brimonidine is believed to have neuroprotective effect, which is an important parameter of glaucoma pathogenesis.

Brimonidine is used as a first-line therapy in patients who have contraindications to β blockers. In a double-masked, placebo-controlled trial, brimonidine was effective in reducing the IOP in patients with elevated IOP. Its efficacy was equivalent to that of apraclonidine.[22] Due to high selective action on a-2 adrenoceptor agonist activity, the IOP-lowering ability of brimonidine may be comparable with that of timolol and dorzolamide and superior to betaxolol.[23–25] Brimonidine is available as 0.2 and 0.5%, to be applied twice daily.

## β BLOCKERS

Since their introduction in 1979, β blockers have become first line therapy for glaucoma. These agents reduce intraocular pressure (IOP), thereby preventing damage to the optic nerve and subsequent loss of vision. Timolol, betaxolol, levobunolol, metipranolol, and carteolol are the topical beta-blockers available in the market. They have similar IOP-lowering efficacy, but differ in other pharmacological properties.

Timolol has become the most widely used ocular hypotensive agent. The potential side effects associated with its nonselective beta-blockade have prevented its use in patients with reactive airways disease and with various cardiovascular conditions. Topically administered β blockers are generally well-tolerated. However, they undergo systemic absorption and can adversely affect cardiovascular and bronchopulmonary function in patients with existing diseases such as heart failure, sinus bradycardia, chronic obstructive airways disease or asthma.

Local adverse effects associated with β -blockers include stinging, burning, red eye, itching, tearing and loss of corneal sensitivity.[26,27]

**Figure 3:**
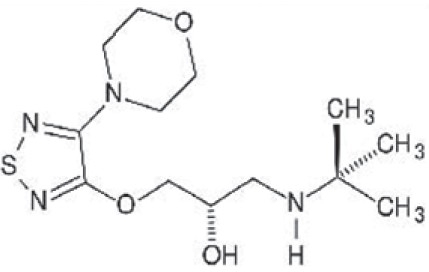
Structure of timolol

Timolol [Figure 3], introduced in 1978, was the first β adrenocpetor antagonist approved for the treatment of glaucoma. It lowers IOP by decreasing aqueous humor formation. It reduces IOP by 20-35%, on an average.[28,29] It is very effective during waking hours and causes less reduction in IOP in night.[30] Early trials demonstrated that it is more effective in lowering IOP, as compared to epinephrine and pilocarpine.[31]

Timolol is available as 0.25% and 0.5% ophthalmic solutions, to be applied twice daily, and also as once-daily gel (0.5%) forming solutions (Timoptic XE). With timolol, systemic complications occur more frequently, including a variety of cardiovascular, respiratory, central nervous system, gastrointestinal, and dermatologic reactions. Ocular side effects were also reported, including superficial punctate keratitis, ocular pain or discomfort, corneal anesthesia, and vague visual disturbances.[32] Timolol is the US-FDA's gold standard drug for glaucoma therapy, against which all new medications must be compared prior to approval.

### Carteolol

**Figure 4:**
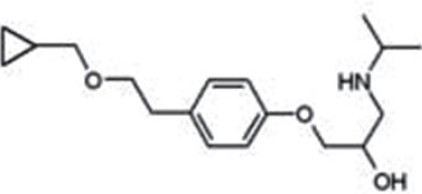
Structure of betaxolol

Carteolol hydrochloride [Figure 4] ophthalmic solution, 1%, is a nonselective beta-adrenergic blocking agent with associated intrinsic sympathomimetic activity. It was hoped that the intrinsic sympthomimetic activity (ISA) of carteolol might protect against some of the systemic adverse effects such as reduced pulse and blood pressure, seen with other beta-adrenoreceptor antagonists. Given topically twice daily in controlled domestic clinical trials, carteolol produced a median percent reduction of IOP 22 to 25%. Carteolol 1% showed comparable ocular hypotensive effect and a safety profile, similar to those of timolol 0.5% solution, and it was better tolerated, with regard to stinging and irritation.[33]

### Levobunolol

Levonunalol acts by reducing aqueous humor formation and increasing outflow facility.[34] The onset of action with levobunalol is seen within one hour and the maximum effect is observed between two and six hours. A majority of it is metabolized into an active metabolite di-hydrolevobunolol, which is also effective at lowering IOP. It is used clinically in a concentration of 0.5 to 1%, twice or once a day.

Betaxolol [Figure 4] is a relatively selective β-1 blocker, which in most patients is almost as effective as timolol in lowering intraocular pressure and may be partly additive with dipivefrin. It is probably safer in patients unable to tolerate non-selective β -blockers.[35]

The onset of action with betaxolol is within 30 minutes and the maximum effect is observed two hours after topical administration. It is available as 0.25% ophthalmic suspension, to be administered twice daily. Stinging upon instillation is a particularly frequent finding with betaxolol (up to 30% to 40% of patients). Unlike other topically applied beta-blockers, betaxolol plays an additional role in blocking N-methyl-Daspartate (NMDA) gated calcium channels.

### Systemic carbonic anhydrase inhibitors

Carbonic anhydrase inhibitors are sulfonamide drugs, which act on the ciliary epithelium, on -carbonic anhydrase isoenzyme II catalyses conversion of CO_2_ and H_2_O to HCO_3_ and H+, a process important for the production of aqueous humor.

Acetazolamide and methazolamide are able to reduce IOP, when taken orally, by decreasing aqueous production. Acetazolamide tablets (125 mg and 250 mg) and methazolamide tablets (25 and 50 mg) are available in the market. Acetazolamide is administered four times daily and methazolamide two or three times daily. Both medications cause several side effects, including paresthesia of fingertips and toes, fatigue, depression, kidney stones, thrombocytopenia, agranulocystosis, and aplastic anemia.[36]

### Topical carbonic anhydrase inhibitors

**Figure 5:**
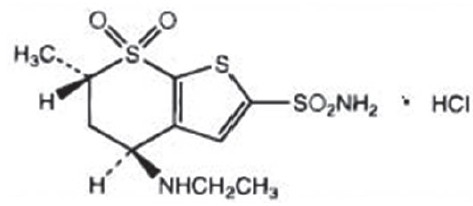
Structure of dorzolamide

Dorzolamide [Figure 5] was the first topical carbonic anhydrase inhibitor launched in the market.

Dorzolamide reduces IOP by decreasing aqueous production, through the inhibition of the enzyme carbonic anhydrase in the ciliary body. When β-blockers are contraindicated, dorzolamide may be used as a first-line therapy. It has excellent additivity with β blockers and pilocarpine.[37]

Dorzolamide is most commonly prescribed as an add-on therapy. It is available as a 2% ophthalmic solution applied three times daily. An IOP reduction of approximately 19-23% is observed.[38]

A combination of latanoprost and dorzolamide showed additive effect in lowering the IOP in a trial with 30 patients of ocular hypertension or early capsular or primary open-angle glaucoma and elevated IOP.[39] A double-masked, randomized one-year study revealed that ocular hypotensive efficacy of 2.0% dorzolamide, given three times a day, is comparable with that of 0.5% betaxolol, given twice daily.[40] Systemic side effects are minimal, as compared with those of oral carbonic anhydrase inhibitors. However, there were local side effects, including corneal edema, borderline endothelial function, decreased visual acuity and allergic reactions. Local adverse events seen with dorzolamide include stinging, burning and itching.

### Brinzolamide

Brinzolamide, available as 1% ophthalmic suspension, has been able to lower IOP as well as dorzolamide. Its pH of 7.4 equivalent to that of human tears makes it better tolerated than dorzolamide (pH 5.5) by most patients.[41,42]

A multicenter, double-masked, prospective, parallel-group study showed that brinzolamide 1.0% caused less ocular discomfort than dorzolamide 2.0%. The incidence of ocular discomfort (burning and stinging) on instillation of brinzolamide (twice daily, 1.8%; three times daily, 3.0%) was significantly less compared with the treatment with dorzolamide (16.4%).[43]

### Prostaglandin analogs

**Figure 6:**
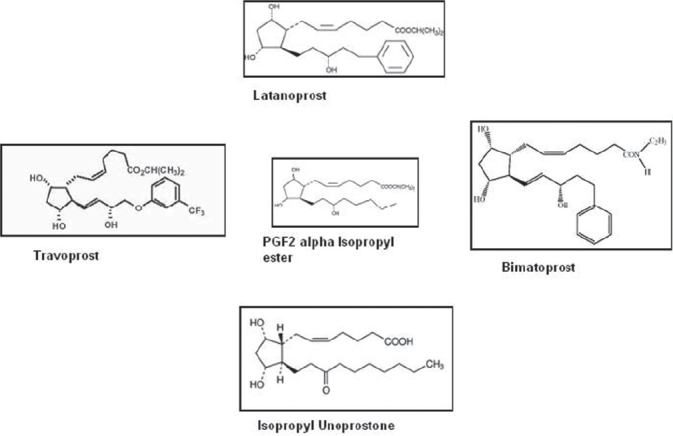
Chemical structures of prostaglandin F2α and commercially available prostaglandin analogues

Prostaglandins (PG) are known mediators of inflammation. At high doses, they can induce increased IOP. Conversely, at low doses prostaglandins have been shown to lower IOP.[44] Hypotensive lipids, named as eicosanoids, including latanoprost, travaprost and bimatoprost. Prostaglandin analogs [Figure 6] represent a novel class of topically active ocular hypotensive agents with a unique mechanism of action.

### Latanoprost

Latanoprost, an ester prodrug analogue of a prostaglandin F2a (13,14-dihydro-17-phenyl-18,19,20-trinor-PGF2a isopropyl ester) analogue, is a selective prostanoid FP receptor agonist. Several clinical trials have demonstrated that it can be combined with timolol, acetazolamide, epinephrine, and pilocarpine, probably due to its unique IOP-reducing mechanism.[45–50]

Latanoprost reduces IOP by increasing the aqueous outflow from the eye, through the uveoscleral pathway.[51] How this occurs is not known, but it is thought that they bind to the receptors of the ciliary body and upregulate metalloproteinases. These enzymes remodel the extracellular matrix and make the area more permeable to aqueous humor, thereby increasing outflow.[52] A single drop of latanoprost 0.005% solution (about 1.5*µ*g) once daily has been established as the most effective dosage regimen.[53]

Since its introduction in 1996 in the US, latanoprost has become the most popular drug for the treatment of glaucoma around the world. Latanoprost has been compared with timolol in several multicentric clinical trials. Once-daily latanoprost was found to be more effective in lowering IOP than twice daily timolol. The mean IOP reduction was 6.7± 3.4 mmHg for latanoprost and 4.9±2.9 mmHg for timolol, after 6 months' treatment.[54–56]

Latanoprost was found to be effective in reducing IOP during the evening as well as during the day.[57] A long-term study of five years with latanoprost has shown no loss in efficacy in treating glaucoma patients, for this study latanoprost was declared as the only PGA to have received a formal first-line usage approval from the US-FDA. In some studies, latanoprost was found to be equally significantly more effective in reducing IOP than dorzolamide and brimonidine.[58] In a comparative study between three PGAs latanoprost, bimatoprost, and travaprost, it was found that all the three drugs were comparable in their ability to reduce IOP in OAG and OH patients. Latanoprost exhibited greater ocular tolerability.[59] Latanoprost is available in 0.005% solution, administered in the evening and requiring refrigeration for long-term storage as well as protection from sunlight and stability.[60]

Conjunctival hyperemia occurs within the first two days after instillation of latanoprost treatment, which diminishes with time (after two to four weeks). Increased iris pigmentation has been reported in 5 to 25% of glaucoma patients treated with latanoprost. Irideal darkening may be a result of a prostaglandin-stimulated increase in melanin production.[61] Several effects on eyelid and lashes were seen, following treatment with latanoprost, including an increase in the length, number, colour and thickness.[62]

Previous surgery or a history of intraocular inflammation may predispose some glaucoma patients treated with latanoprost to cystoid macular oedema or uveitis. Systemic adverse effects are relatively not seen because the drug and its metabolites have rapid elimination half-life.

[Figure 6]: Chemical structures of prostaglandin F2a and commercially available drugs belonging to prostaglandin analogues

### Unoprostone

Unoprostone Isopropylate is a docosanoid, a structural analogue of an inactive biosynthetic cyclic derivative of arachidonic acid, 13, 14-dihydro-15-keto-prostaglandin F2a. Its chemical name is isopropyl (+)-(Z)-7-[(1R, 2R, 3R, 5S)-3,5-dihydroxy-2-(3oxodecyl) cyclopentyl] -5-heptenoate. It differs structurally from other PGAs in that it has a 22-carbon chain backbone, instead of the typical truncated 20-carbon structure found in other agents [Figure 1] It is available in 0.15% ophthalmic formulation, to be applied twice daily.

Unoprostone decreases IOP by increasing the outflow facility without affecting aqueous humor production.[63] When used in monotherapy, Unoprostone provided a clinically significant IOP-lowering effect, equivalent to that of betaxolol but not to those of timolol and latanoprost.[64,65] However, in another study, an aqueous solution of 0.12% unoprostone isopropyl, applied topically to the eye twice daily for six weeks, was as effective as 0.5% timolol in maintaining control of IOP in subjects with chronic open angle glaucoma or ocular hypertension.[66] In a six-month study, it was found that unoprostone isopropyl beneficially provides additive IOP lowering effect to topical β-blocker in patients with primary open angle glaucoma. No serious systemic side effects were found in the present study.[67]

A study involving thirty healthy volunteers, Unoprostone significantly increased microcirculation in the optic nerve head (ONH) in control subjects and in normal tension glaucoma patients, without reducing the IOP significantly.[68] A long term comparative study between topical antiglaucoma therapy of timolol and unoprostone as against betaxolol and unoprostone revealed that both combined treatments were effective for IOP reduction in glaucoma patients, and the data from the Betaxolol and Unoprostone treatment group suggested that Betaxolol and Unoprostone was more effective in maintaining visual field than timolol and unoprostone.[69] Unoprostone instillation increases blood flow in the choroidretina in human eyes.[70] Iris hyperpigmentation and abnormal eyelash changes may occur after treatment with unoprostone, but the incidence of these events were low in the two-year clinical study.[71]

### Bimatoprost

Bimatoprost ophthalmic solution 0.03% is a synthetic prostamide analog with ocular hypotensive activity. Its chemical name is (Z)-7-[(1R,2R,3R,5S)-3,5-dihydroxy-2[1E,3S) -3-hydroxy-5-phenyl-1-pentenyl]cyclopentyl]- 5-N-ethylheptenamide.

Bimatoprost is known as prostamide analog, because of the unique structural presence of an amide ester group at the carboxy terminal end of the a carbon chain [Figure 6].

Bimatoprost interacts with a prostamide receptor in the trabecular meshwork, to increase outflow facility.[72] Bimatoprost enhances the pressure-sensitive outflow pathway and may also cause an increase in the rate of flow via the pressure-insensitive outflow pathway and a lowering of the extraocular recipient pressure.[73]

In a six-month multicentric, randomized controlled trial, bimatoprost proved to be statistically and clinically superior to timolol in lowering IOP in patients with glaucoma or ocular hypertension. The most frequent side effect was trace-to-mild conjunctival hyperemia. Changes in iris pigmentation were reported in 1.1% of bimatoprost patients.[74]

A multicenter, randomized, investigator-masked, parallel-group trial bimatoprost provided lower mean pressures than latanoprost at every time point throughout the study and was statistically superior in achieving low target pressures.[75] A six-month trial study revealed that the IOP-lowering efficacies of bimatoprost and timolol-dorzolamide combination were similar; thus, bimatoprost can be used as a long-term monotherapy agent in the treatment of POAG and ocular hypertension.[76]

Bimatoprost is available in 0.03% ophthalmic solution and is administered once daily in the evening. It does not require refrigeration to maintain stability.[77]

### Travaprost

Travaprost is a synthetic prostaglandin F 2a analogue. Its chemical name is isopropyl (Z)-7- [(1 R,2 R,3 R,5 S)-3,5-dihydroxy-2-[(1 E,3 R)-3-hydroxy-4-[(a,a,a-trifluoro- m -tolyl)oxy]-1- butenyl]cyclopentyl]-5-heptenoate. Following absorption into the eye, the free acid form of travaprost interacts with the endogenous FP prostanoid receptor, to enhance aqueous humor outflow and lower intraocular pressure (IOP). It differs from other PGAs, which exhibit partial agonist activity, in that it is a full agonist at the PGF2a receptor.[78]

In clinical studies, travaprost once daily produced reductions in IOP of between 7-8 mmHg, from a mean baseline IOP of 25-27 mmHg, an effect similar to that noted in the case of bimatoprost or latanoprost.[79] In controlled clinical trials, travaprost 0.004% once daily, used as monotherapy, produced greater IOP reduction than timolol 0.5% b.i.d and equal or greater reduction than latanoprost 0.005%.[80]

Travaprost 0.004% was also shown to be an effective adjunctive agent, offering an additional 5 - 7 mmHg IOP reduction in patients inadequately controlled on timolol 0.5%. In trial, it was observed that travaprost monotherapy had better lowering than dorzolamide 2.0% timolol maleate 0.5% fixed combination.[81]

Travaprost provides robust lowering of IOP with little diurnal fluctuation and results in low target pressures in a large percentage of the patients.[82] It is very stable compound, to be applied once daily in the evening. It does not require refrigeration and protection from sunlight.[83]

Macular oedema, including cystoid macular oedema, is cited as a warning in the US product labeling for travaprost, as it is for other prostaglandin analogues.

### Combination therapy

When a single therapy is not sufficient to lower the IOP, a combined treatment is indicated. The combination therapy is also dependent upon the mechanism through which the components act to reduce IOP. When choosing an agent for combination therapy, it should be borne in mind that those drugs with complementary mechanisms of action usually work together. Fixed-combination products have the combined efficacy of two ocular hypotensive drugs and the convenience of a two-drug treatment regimen in a single container, which may aid patient adherence to treatment. If a beta-blocking agent is used as an initial treatment, adding a topical CAI can provide an additional reduction in IOP.[84] Another combination product comprises 0.005% latanoprost and 0.5% timolol. The addition of latanoprost to timolol treatment produces an additional IOP reduction of 13-37%, depending upon the frequency of the application and the baseline IOP. Available fixed-combination products consist of timolol 0.5% as an invariant, with brimonidine 0.2%, dorzolamide 2%, travaprost 0.004%, latanoprost 0.005% or bimatoprost 0.03%.[85]

The range of reported additional reductions in IOP, compared to a latanoprost monotherapy baseline are as follows: latanoprost-timolol (13-37%), latanoprostpilocarpine 2% (7-14%), latanoprost and carbonic anhydrase inhibitors (15-24.1%), and latanoprost and dipivefrin (15-28%).[86]

Brimonidine 0.2% combined with 0.5% timolol is the newest fixed combination product in the market. Clinical trials have demonstrated that dorzolamide/timolol (1 drop per eye twice daily) is an effective and generally well-tolerated fixed combination for lowering IOP in patients with open angle glaucoma or OH, including individuals uncontrolled on β -adrenoceptor antagonist monotherapy.[87]

### Laser procedures

A secondary choice of treatment of glaucoma is the use of laser therapy. The primary strategy involves ‘burning’ holes in various areas within the eyes, including the ciliary and the pigmented trabecular meshwork cells.[88]

Argon laser trabeculoplasty (ALT) targets trabecular meshwork, where it allows the aqueous fluid to leave the eye more efficiently. The procedure requires 10-20 minutes and 80% of the patients respond well to it and may eventually discontinue glaucoma medications.[89]

The Nd : YAG (neodymium-doped yttrium aluminium garnet) laser can also be used in closed-angle glaucoma to make a small peripheral hole in the iris, to allow the aqueous fluid to flow easily. Selective laser trabeculoplasty (SLT) delivers energy to pigmented trabecular meshwork cells in a process called photo-thermolysis. The advantage of SLT is that nonpigmented trabecular meshwork (TM) cells may sustain less damage compared with ALT.[90]

### Surgery

Trabeculectomy should be considered in all patients, when the ‘target IOP’ is not achieved with glaucoma medications and if the expected rate of visual loss could affect the patient during their lifetime. In this procedure, an opening is made in the trabecular meshwork, so that aqueous humor can drain into the sclera.

Many patients can discontinue glaucoma medications after surgery. Approximately one-third of the trabeculectomy patients develop cataract within five years. If trabeculectomy fails, another type of surgery places a drainage tube (Molteno tube) in the eye, between the cornea and iris, which exits at the junction of the cornea and sclera. Cyclodestructive procedures, which lower IOP by destroying the ciliary body, are typically reserved for eyes, which are refractory to all other forms of therapy. These procedures include cyclocryotherapy, cylcodiathermy and laser cyclophotocoagulation.[91–93]

### Complementary and alternative system of medicine

In recent times, there has been an increased interest in complementary medicine. But very little research has been done on the majority of herbal remedies, with regard to their effect on glaucoma. There are several nutrients and botanicals that hold promise for the treatment of glaucoma, but most studies are preliminary, and larger, controlled studies are required.[94]

Forskolin is a diterpine derivative of the plant Coleus forskohlii, which acts on adenylate cyclase catalytic subunit to increase intracellular cAMP. Gingko biloba extract has multiple beneficial actions, which will be helpful in the treatment of glaucoma, like increased ocular blood flow, antioxidant activity, platelet activating factor inhibitory activity, nitric oxide inhibition, and neuroprotective activity combine, suggesting that Gingko biloba extract could be used in the treatment of glaucoma.[95]

In a recent trial in Italy, which was a randomized, placebo-controlled, double masked trial involving 27 patients, there was an improvement in the visual fields in-patient, with normal tension glaucoma after four weeks of treatment with Ginkgo biloba.[96]

Alpha lipoic acid, a powerful antioxidant, may be useful in glaucoma, because it reduces nerve cell damage from oxidative stress.[97] Supplementation with Vitamin C is believed to increase aqueous humor drainage, through reducing the viscosity of hyaluronic acid in the trabecular meshwork.

In China, the main herb-derived eye drops for glaucoma are pueraria flavonoids, areca seed extract, and alkaloids from erycibe (Erycibe obtusifolia; dinggongteng aka baogongteng). These eye drops appear to work as well or better than pilocarpine, which is usually used as a comparative standard.[98] Salvia miltiorrhiza is a Chinese herb, injected intravenously (solution) and which helps to improve microcirculation of the retinal ganglion cells.

Cannabinoids reduce intraocular pressure by enhancing uveoscleral outflow. The development of formulation for ocular administration has not yet yielded a prescription medication.

### Future glaucoma therapy

A number of potential strategies for the development of a novel therapy for glaucoma are: glutamate inhibition, NMDA receptor blockade, exogenously applied neurotrophins, open channel blockers, antioxidants, protease inhibitors and gene therapy.

### NMDA receptor antagonists

NMDA antagonist provides neuroprotection by blocking pathological increase in glutamate, which drives cell death by facilitating calcium entry into a cell. Memantine, an N-methyl-D-aspartate subtype glutamate receptor antagonist is in the clinical stage of development, and, if there is proof of efficacy of memantine, it will change the treatment paradigm for glaucoma.[99]

In addition to memantine, a number of other potential non-IOP lowering direct acting neuroprotective agents are shown to have an application in glaucoma. Many of these agents focus on other routes of overcoming glutamate cytotoxicity.

Compounds on clinical trials are –

Eliprodil: It is a non-competitive NMDA antagonist; providing protection from glutamate mediated cytotoxicity to retinal ganglion cells.

Riluzole: It is a presynaptic glutamate release inhibitor, which has shown to have potential neuroprotective utility.

L-deprenyl: An inhibit apoptosis of serum deprived retrovirus-immortilised retinal ganglion cells in vivo, it can decrease the apoptosis index of primary mix retinal cells, when deprived of specific neurotropic factors.

### Neuroprotective vaccines

Since resistance to high IOP is immune-dependent, T-cell induced neuroprotection may vaccinate the RGC from apoptosis. An example of a neuroprotective vaccine is R16, a peptide (interphotoreceptor-retinoid binding protein) derived from the RGC.[100]

### STAT-3 activation

Signal transducers and activators of transcription protein-3 (STAT-3) play an important role in cell growth and differentiation. They are considered important because the mRNA of this protein is upregulated in rats with glaucoma. Ciliary neurotrophic factor (CNTF), which is an interleukin-6 cytokine injected into the eyes of rats with increased IOP reduced apoptosis, phosphorylated STAT-3, and reduced the activity of caspase-3. Interleukin-10 also has neuroprotective activity, which promotes survival of RGCs, due to IL-10 signaling through the STAT-3 pathway.[101–103]

### Erythropoietin

Erythropoietin is a hematopoietic cytokine, which has been shown to possess remarkable tissue-protective and neuroprotective properties that may prevent further RGC death by inhibiting apoptosis.[104] Intravitreal injection of Erythropoietin in rats with axotomized RGCs enhances RGC survival by 92%, as compared to those without EPO injection.[105] In addition, EPO reduces caspase activity, indicated by a decrease in the absorbance of colorimetric caspase substrates. However, further studies are needed to fully evaluate the safety and efficacy of this neuroprotective agent in clinical trials.[106]

### Caspase inhibitors

Gene therapy represents an attractive approach for the treatment of eye diseases such as glaucoma. Inhibitors of apoptosis protein (IAP) can also reduce apoptosis by inhibiting caspase. Ocular administration of viral vectors produces localized retinal gene expression with reduced risks of side effects reported with systemic administration of viral vectors. Rats were given unilateral intravitreal injections of AAV-CBA vector coding for human baculoviral IAP repeat-containing protein-4 (BIRC4), a potent caspase inhibitor. Gene therapy delivering BIRC4 significantly promoted optic nerve axon survival in a chronic ocular hypertensive model of rat glaucoma. Blocking RGC apoptosis with caspase inhibitors represents a promising approach for treatment of human glaucoma.[107,108]

### iNOS-2 Inhibitors

Since the upregulation of iNOS-2 is harmful to neurons, its inhibition might have a neuroprotective effect. Although an unregulated level of NO can cause neuronal degeneration via apoptosis, a small amount of NO could inhibit apoptosis. Survival of serum deprived pheochromocytoma PC-12 cells was observed when treated with an NO donor. These results indicate that the cytoprotective effect of nipradilol in PC12 cell death was due to the caspase-3 inhibition mediated by NO-related S-nitrosylation and activation of protein kinase G.[109]

## Conclusions

Glaucoma is a very serious eye disease that can lead to blindness if not treated early. With early diagnosis and treatment, most patients with glaucoma can have their vision restored and enjoy a healthy life. The wide variety of topical effective antiglaucoma drugs that are available today, and a few others that are in the development stage, represent significant advancement in ocular therapeutics. Though these topical ophthalmic preparations have reduced the risk of systemic toxicity to quite an extent, their long-term use causes systemic as well as ocular toxicity. Ideal drug candidates for glaucoma therapy will offer better IOP lowering efficacy with fewer side effects and provide additional means of vision sparing through direct protection of optic nerve. Despite new advances and techniques, it is observed that there is medically uncontrolled intraocular pressure. The ideal medication for this is not yet available. A patient with asthma, bradycardia, hazel eyes, cataracts, systemic allergy to sulfa drugs, and topical allergy to brimonidine might have to proceed with laser trabeculoplasty or glaucoma-filtering surgery.

Research on more advanced antiglaucoma medications continues and promising new directions appear to be the Rho-kinase inhibitors, microtubule-disrupting agents, serotonergics and cannabimimetics. The research is being directed towards applying new molecular and cellular techniques to induce regeneration of mammalian central nervous axons. This will be an important step in therapy for glaucomatous optic nerve atropy.
